# Loss of Robustness and Addiction to IGF1 during Early Keratinocyte Transformation by Human Papilloma Virus 16

**DOI:** 10.1371/journal.pone.0000605

**Published:** 2007-07-11

**Authors:** Tamar Geiger, Alexander Levitzki

**Affiliations:** Unit of Cellular Signaling, Department of Biological Chemistry, The Alexander Silverman Institute of Life Sciences, The Hebrew University of Jerusalem, Jerusalem, Israel; University of Hong Kong, China

## Abstract

Infection of keratinocytes with high risk human Papilloma virus causes immortalization, and when followed by further mutations, leads to cervical cancer and other anogenital tumors. Here we monitor the progressive loss of robustness in an in vitro model of the early stages of transformation that comprises normal keratinocytes and progressive passages of HPV16 immortalized cells. As transformation progresses, the cells acquire higher proliferation rates and gain the ability to grow in soft agar. Concurrently, the cells lose robustness, becoming more sensitive to serum starvation and DNA damage by Cisplatin. Loss of robustness in the course of transformation correlates with significant reductions in the activities of the anti-apoptotic proteins PKB/Akt, Erk, Jnk and p38 both under normal growth conditions and upon stress. In parallel, loss of robustness is manifested by the shrinkage of the number of growth factors that can rescue starving cells from apoptosis, with the emergence of dependence solely on IGF1. Treatment with IGF1 activates PKB/Akt and Jnk and through them inhibits p53, rescuing the cells from starvation. We conclude that transformation in this model induces higher susceptibility of cells to stress due to reduced anti-apoptotic signaling and hyper-activation of p53 upon stress.

## Introduction

Cancer development is a multi-step process in which cells gain high proliferation rates and resistance to apoptotic signals. In this long process cells accumulate mutations and chromosomal aberrations due to impaired cell cycle control and DNA repair mechanisms. In the past few decades, thousands of research projects have characterized tumor suppressors and oncogenes, which have abnormal activities in human cancers. Two tumor suppressors, p53 and Rb, have been shown to be inactive in the vast majority of cancers. Their inactivation impairs cell cycle arrest and apoptosis in response to cell damage. On the other hand, the enhanced activities of oncogenes, such as receptor tyrosine kinases, among them the IGF1 receptor, induce higher proliferation rates and resistance to apoptotic stimuli. Much less effort has been devoted to characterizing the initial steps of transformation. This is mostly due to the lack of a cell system that encompasses the continuum from the normal cell through full transformation. When comparisons are made, they refer to the fully-transformed tumor cell in comparison to its normal untransformed counterpart.

Human Papilloma virus, primarily types 16 (HPV16) and 18 (HPV18), are considered to be the causative agents for cervical cancer and other anogenital lesions [1]. Infection induces immortalization of cells, mainly as a result of activation of two viral genes: E6 and E7. The E6 protein targets p53 for proteasomal degradation, and E7 targets Rb for proteasomal degradation [2]–[6]. Thus the E6 and E7 proteins interfere with cell cycle control and apoptosis, and induce genomic instability in cells [7]. HPV infection is responsible for cell immortalization, but full transformation and tumor formation occur only years after infection. During the period between infection and cancer development, the cells accumulate mutations, which eventually cause full transformation.

In this study we monitor changes in the early stages of transformation, before cancer development. We have previously suggested that transformed cells are more sensitive to stress, which may partially account for their enhanced sensitivity to cytotoxic agents [8], [9]. In that study, we compared immortalized NIH3T3 cells to immortalized NIH 3T3 cells expressing oncogenes. In the current study we have monitored the progressive sensitization to stress upon the immortalization of normal cells and during the initial steps of transformation, characterizing the changes in the pre-malignant stage. Using an in-vitro cellular model system for the development of cervical cancer, we have correlated the gradual development of sensitivity to stress with the degree of cell transformation. We examined HPV16-immortalized keratinocytes extensively passaged in vitro, HF1 cells, comparing cells from early and late passages. In late passages cells gain higher proliferation rate and acquire the ability to form colonies in soft agar, but still do not form tumors in nude mice, therefore have not reached full transformation. Using this model of progressive cellular transformation, we could compare immortalized cells from various stages with their normal counterpart, namely, uninfected human keratinocytes. When comparing normal keratinocytes to cells in early passage (∼60 doublings) after HPV16 transfection, we are monitoring early events caused by the expression of HPV16 genes. The differences between late passages (∼1000 doublings) and early passages most likely result from additional genetic and epigenetic changes in these cells. We find that sensitivity to serum starvation and to Cisplatin (CDDP) treatment correlates with the number of passages. Moreover, as transformation proceeds in our model, there is a reduction in the activity of key anti-apoptotic proteins, PKB/Akt, Erk, Jnk and p38, and increased activation of p53 upon stress. During starvation the transformed cells become dependent on IGF1, which activates the above mentioned kinases, and inhibits p53. Treatment with IGF1 rescues the cells via activation of PKB/Akt and Jnk, and through them, inhibition of p53 induction. Similar results were obtained using an independent line of HPV16-immortalized keratinocytes, HPKII cells, but not with the fully transformed SiHa line. Sensitivity to stress correlates with high induction of p53 activity, which is reduced with IGF1 treatment and activation of PKB/Akt. We conclude that during early stages of cellular transformation, cells gain higher proliferation rate, which occurs concurrently with higher activation of p53 and a decrease in cell robustness.

## Methods

### Experimental Procedures

#### Materials

Cell culture reagents were purchased as follows: DMEM, HAM/F12, RPMI and antibiotics from Biological Industries Israel Beit Ha'emek Ltd.; FBS from Gibco BRL; EGF from Peprotech Inc.; insulin, T3, transferrin, cholera toxin and hydrocortisone from Sigma. Signal transduction inhibitors were purchased as follows: Akt inhibitor V (triciribine), PD98059, SP600125, SB203580, U0126 from Calbiochem; Cisplatin was kindly received from Hadassah Hospital; Growth factors were purchased as follows: IGF1, EGF and LPA from Sigma and NRG from R&D Systems.

### Cell culture

Primary keratinocytes and HF1 cells were grown in keratinocyte growth medium (67% DMEM, 23% HAM/F12, 10% FBS, 5 µg/ml insulin, 2 nM T3, 5 µg/ml transferrin, 0.4 µg/ml hydrocortisone, 0.1 nM cholera toxin, 10 ng/ml EGF and antibiotics). Primary keratinocytes were taken from small biopsy specimens from control healthy donors. Cultures were kindly provided to us regularly by Professor H. Ben-Bassat (Hadassah University Hospital, Jerusalem, Israel). Primary keratinocytes from second or third passage were used in the experiments reported here. HF1 cells were produced by transfection of the whole HPV16 genome to human foreskin keratinocytes [10], [11]. HF1 cells from various passages were also obtained from Professor H. Ben-Bassat. Early HF1 cells are ∼60 doublings after HPV16 transfection. Late HF1 cells are ∼1000 passages after transfection.

HPKII cells were kindly provided to us by Professor M. Duerst (Friedrich-Schiller-Universität, Jena, Germany). HPKII cells were grown in DMEM (low glucose) containing 10% FBS and antibiotics. HPKII cells were generated by transfection of HPV16 genome into human foreskin keratinocytes [12], [13] . Early HPKII cells are ∼70 doublings after transfection, and late HPKII cells are ∼300 doublings after transfection.

SiHa cells were kindly provided to us by Professor S. Rosenbaum-Mitrani (Hadassah Hospital, Jerusalem, Israel). SiHa cells were grown in RPMI containing 10% FBS and antibiotics.

### Methylene Blue assay

Primary keratinocytes, early HF1 cells and late HF1 cells were plated in 96-well plates at a density of 2500 cells per plate. Every 24 h a representative plate was fixed with 0.5% gluteraldehyde. Methylene blue assay was performed as described elsewhere [10].

### Soft agar assay

Primary keratinocytes, early HF1 cells and late HF1 cells were plated in agar at a density of 5000 cells per well in a 96-well plate in growth medium containing 0.3% agar (50 µl per well), on top of a layer of growth medium containing 1% agar (100 µl per well). 50 µl of growth medium were added on top of the agar. Two weeks after plating, the cells were stained with 3-(4,5-dimethylthiazol-2-yl)-2,5-diphenyl-tetrazolium bromide (MTT; Sigma) and photographed.

### Cell cycle analysis

HF1 cells, HPKII cells and SiHa cells were plated in 6-well plates at a concentration of 1.5×10^5^ cells per well. Primary keratinocytes were plated in 6-well plates at a concentration of 2.5×10^5^ cells per well. Cells were grown in starvation medium (75% DMEM, 25% F12 and antibiotics) or exposed to 20 µM CDDP in the presence or absence of IGF1, growth factors or inhibitors, as indicated in each experiment. After 48 h cells were harvested and fixed with methanol. Fixed cells were rehydrated with PBS, treated with RNase A (5 µg/ml) and stained with propidium iodide (25 µg/ml). Cell cycle analysis was performed by flow cytometry.

### Immuno-blotting

Primary keratinocytes, HF1, HPKII and SiHa cells were plated in 6-well plates as indicated above. 48 h after plating, cells were washed and grown in starvation medium (75% DMEM, 25% HAM/F12 and antibiotics). Where indicated, the cells were stimulated with 50 ng/ml IGF1 for the whole 24 h starvation period or for the last 10 min of starvation. 24 h after initiation of starvation, cells were lysed in sample buffer. Equal amounts of protein from each sample were resolved by PAGE and analyzed by western blot. The following antibodies were used: Monoclonal anti-E7, a kind gift from Dr. Martin Muller, DKFZ, Heidelberg, Germany; anti-PARP, anti-phospho-Akt(T308), anti-phospho-JNK(T183,Y185), anti-phospho-p38(T180,Y182) from Cell Signaling Technology; anti-phospho-Erk(T183,Y185), anti-Tubulin from Sigma; anti-Rb from Pharmingen; anti-Akt1/2, anti-Erk2, anti-JNK, anti-p38 and anti-p53(FL393) from Santa Cruz; anti-beta-Catenin from BD Transduction.

### Immuno-staining

HF1 cells and primary keratinocytes were plated on coverslips in 24-well plates at a concentration of 3×10^4^ cells/well and 5×10^4^ cells/well respectively. 48 h after plating, cells were washed and grown with starvation medium for 12 h, followed by fixation with 3% paraformaldehyde containing 0.5% TritonX100 for 30 sec and with 3% paraformaldehyde for 30 min. Cells were stained with anti-cytochrome C antibody (Santa Cruz).

### Real-time PCR

Primary keratinocytes, HF1, HPKII and SiHa cells were plated in 6-well plates as indicated above. Where indicated, cells were serum starved, treated with IGF1 or treated with IGF1 plus 10 µM Akt inhibitor V or 10 µM SP600125. RNA was isolated from cells with the RNeasy-RNA isolation kit (Qiagen), and subjected to reverse-transcription with random hexamers using ImpromII reverse-transcriptase (Promega) according to the manufacturer's directions. Real-time PCR was performed with the Power Sybr-Green PCR mix, according to the manufacturer's directions. NOXA primers used were: fwd: 5′GCAGAGCTGGAAGTCGAGTGT, rev: 5′AAGTTTCTGCCGGAAGTTCAG HPRT primers used were: fwd: 5′TGACACTGGCAAAACAATGCA, rev: 5′GGTCCTTTTCACCAGCAAGCT.

### Reporter assays

Keratinocytes and HF1 cells were grown in 6-well plates as indicated above. 48 h after plating cells were transfected with LipofectAmine Plus reagent (Invitrogen) according to the manufacturer's directions. Cells were transfected with pRGC-luciferase or pRGC(mutant)-luciferase encoding firefly-luciferase under the regulation of a promoter containing 17 repeats of the p53 response element or of a mutant response element respectively. Both constructs were kindly received from Professor M. Oren (Weizmann Institute of Science, Rehovot, Israel). Cells were co-transfected with a CMV-renilla construct for normalization to transfection efficiency. 24 h after transfection cells were serum starved, and 24 h afterwards cells were lysed with passive lysis buffer (Promega). Firefly and renilla luciferase assays were performed with a dual luciferase assay kit (Promega).

## Results

### Initial transformation is characterized by accelerated growth and anchorage-independent growth

**Figure 1:**
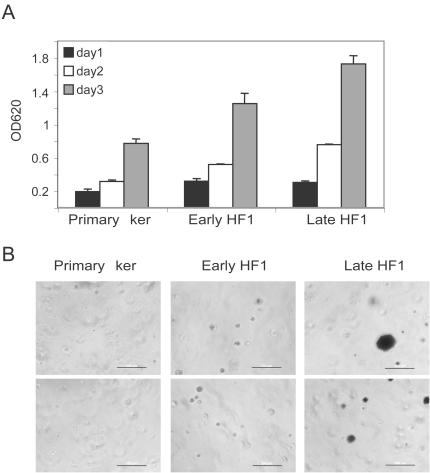
Early transformation induces higher growth rate and enables growth in soft agar. A. Methylene blue assay for cell growth. Primary keratinocytes, early and late HF1 cells were plated at equal densities and grown for three days. Each day a representative plate was fixed. All plates were stained with methylene blue as described elsewhere [10]. Error bars represent standard deviations of triplicates. Similar results were obtained in at least 5 independent experiments. B. Cells were plated at equal densities in soft agar. Two weeks later, live cells were stained with MTT. The figure shows two fields of each cell type (Bar = 200 µm). Similar results were obtained in three independent experiments.

In order to understand the early events that occur during transformation, we studied an in vitro model of keratinocyte transformation with HPV16. We compared primary human keratinocytes to HPV16 transformed cells, HF1 cells, from early stages (∼60 doublings post transfection) and late stages (∼1000 doublings post transfection). As a first step in cell characterization we monitored the growth rates of the various cell types by methylene blue assay. As transformation proceeded growth was accelerated and doubling time was reduced from >24 h in the primary keratinocytes to 22 h in early HF1 and 16 h in late HF1 cells (Figure 1A). Additionally, early in transformation cells gained the ability to survive and grow in soft agar. Primary keratinocytes, early and late HF1 cells were plated in soft agar and grown for two weeks, followed by staining of live cells with MTT. In contrast to the primary keratinocytes, immortalized HF1 cells survived in soft agar. Early HF1 cells survived, but did not proliferate and did not form colonies in soft agar, and are therefore seen as MTT-stained single cells. In contrast, late HF1 cells formed both micro-colonies and more rarely large colonies in soft agar (Figure 1B). Introduction of late HF1 cells into nude mice did not result in tumor formation, so they are not considered fully transformed cells. Therefore higher proliferation rate and growth in soft agar are first steps in cancer progression to full malignant phenotype.

### Sensitivity to stress signals increases in the course of transformation

**Figure 2:**
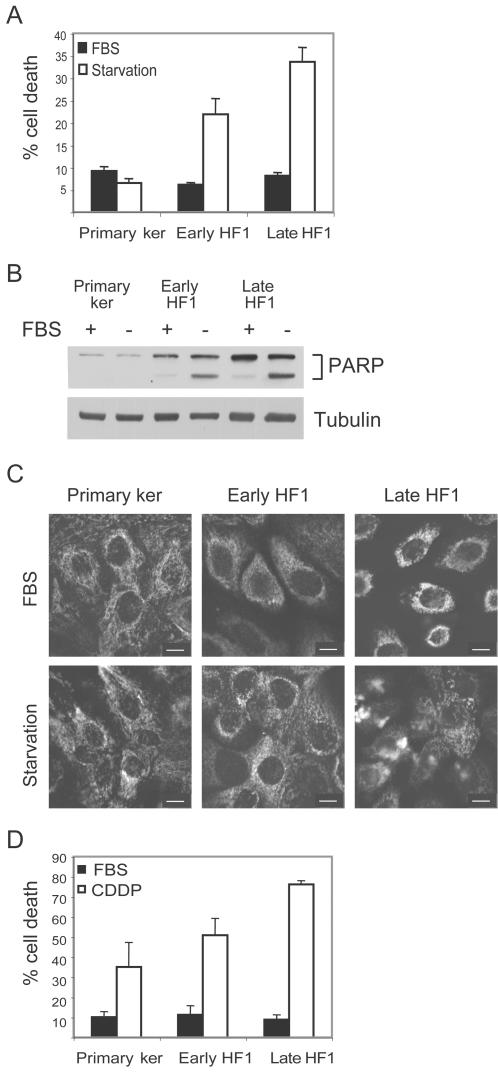
Transformed cells are more sensitive to stress than normal primary keratinocytes. A. Cells were serum starved (white) or untreated (black) for 48 h. After treatment, cells were fixed, stained with propidium iodide and analyzed by flow cytometry. The sub-G1 fraction of the cells was quantified as a measure of cell death. Error bars represent the standard error of three independent experiments. B. Representative western blot of cells after 24 h starvation. PARP cleavage reflects caspase activation during apoptosis. Tubulin serves as a loading control. C. Immuno-staining of cytochrome C after 12 h serum starvation (Bar = 15 µm). D. Cells were treated with 20 µM Cisplatin for 48 h, fixed and analyzed as in A. Error bars represent the standard error of three independent experiments.

In order to investigate whether early transformation induces higher resistance to apoptotic stimuli, we tested the sensitivity of the different cultures to serum starvation. Cells were stained with propidium iodide and submitted to FACS analysis. The sub-G1 fraction was quantified as a measure of cell death. Serum deprivation for 48 h did not induce any increase in cell death of the primary keratinocytes, but rather slightly reduced it (Figure 2A). In contrast, serum withdrawal induced 22% death in early HF1 cells, and 32% death in the more transformed, late HF1 cells. Western blot analysis of cells after 24 h serum starvation and reaction of the blots with anti-PARP antibody showed that cells died via apoptosis. In primary keratinocytes, which were not affected by starvation, PARP was not cleaved. PARP was clearly cleaved upon starvation of early HF1 cells and even more dramatically in late HF1 cells (Figure 2B). We also immuno-stained cytochrome C to monitor its release from the mitochondria. We already detected cytochrome C release after 12 h of starvation in late HF1 cells, but not in primary keratinocytes or early HF1 cells, which are more resistant to the stress (Figure 2C). In order to see whether the increased sensitivity to stress was a more general phenomenon or restricted to starvation, we exposed the cells to the DNA damaging agent, Cisplatin. Cisplatin treatment induced cell death in all three cell types and again, sensitivity increased with transformation (Figure 2D). Treatment with 20 µM Cisplatin for 48 h induced 35% death of normal keratinocytes, 50% death of early HF1 and ∼80% death of late HF1 cells.

These experiments show a clear increase in sensitivity to stress as a result of cell immortalization and in vitro passaging. Stress induced faster activation of mitochondrial apoptosis, which results in caspase activation and DNA fragmentation. The fact that only HF1 cells, but not the primary keratinocytes, were sensitive to serum starvation provided a convenient, clear-cut system for further analysis. We therefore concentrated our studies on the response of HF1 cells to serum starvation.

### The involvement of p53 in Rb in the response to stress

**Figure 3:**
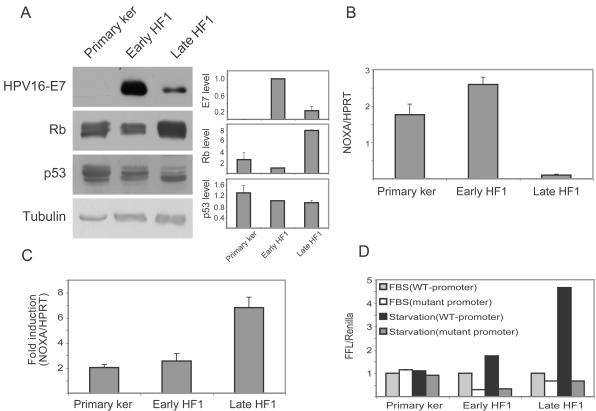
P53 and Rb under basal conditions and during starvation. A. Representative western blots of whole cell lysates of primary keratinocytes, early and late HF1 cells under basal growth conditions. Blots show the status of HPV16 E7 protein, Rb and p53. Graphs show quantification of these blots. Protein densities were normalized to the densities of the bands in early HF1 cells. Error bars represent the standard deviations of three independent experiments. Tubulin serves as a loading control. B. Real-time PCR for NOXA expression under basal conditions. RNA was isolated from untreated cells and subjected to reverse-transcription followed by real-time PCR with specific primers for NOXA and HPRT as control. NOXA results were normalized to the HPRT results. Error bars represent the standard errors of three independent experiments. C. Real-time PCR for NOXA expression in starved vs. untreated cells. Cells were serum starved for 24 h, RNA was isolated, reverse-transcribed and subjected to real-time PCR as in B. Fold induction represents results of starved cells vs. non-starved cells. Error bars represent the standard error of three independent experiments. D. Reporter assay for p53 activity in untreated and serum starved cells. Cells were transfected with firefly-luciferase (FFL), either under the control of 17 p53-response elements, or under the control of mutant response elements as control. For transfection control cells were co-transfected with renilla-luciferase under the control of CMV promoter. 24 h after transfection cell medium was replaced with normal growth medium or starvation medium, for another 24 h. Cells were lysed and subjected to luminisence assay. Error bars represent standard errors of four independent experiments.

It has been shown that expression of the E6 or E7 genes of HPV often increases stress sensitivity [14]–[17]. Since transformation in our system initiated with HPV16 transfection, we examined the expression of the viral genes and their targets, p53 and Rb, to see whether the increased stress sensitivity could be attributed to their expression. Since there are no efficient antibodies against E6, and the E6 and E7 genes are co-regulated, E7 expression is a valid indicator for viral gene expression. The E7 protein was very highly expressed in early HF1 cells, and its level was markedly reduced in late HF1 cells (Figure 3A). Our results suggest that the viral proteins trigger the initiation of transformation, but their effects are reduced in later stages. In agreement with the expression of E7, the Rb protein level was reduced in early HF1 cells, but elevated in late HF1 cells. Thus Rb expression did not correlate with sensitivity to stress.

The level of p53 was gradually reduced upon progression from primary keratinocytes, through early to late HF1 cells (Figure 3A). P53 appeared in the western blot as three bands; the lower band was intensified in late HF1 cells, but the other two almost disappeared. Sequencing of p53 cDNA in these cells revealed no mutation or deletion in the p53 coding sequence (data not shown), so the three bands appear to represent wt p53 with different post-translational modifications. The p53 protein is very highly regulated, mainly via phosphorylation and proteasomal degradation [18]. Reaction with several antibodies that recognize different phosphorylated forms of p53 did not shed light on the protein's activity in the various cell types (not shown). In order to compare p53 activity in the three cell types under normal growth conditions we looked at the expression of p53 targets. Table 1 shows expression levels of six known p53 targets, which were obtained from Affymetrix micro-arrays (Kravchenko-Balasha, in preparation). There was a gradual decrease in the expression of GADD45, p21, MMP2 and TGFalpha from the primary keratinocytes through early HF1 cells, to late HF1 cells. NOXA and IGFBP3 expression rose slightly in early HF1 cells, but was dramatically reduced in the later stage. GAPDH expression is given as an internal control for constant mRNA expression. NOXA is a known mediator of the anti-apoptotic effects of p53 [19]. We therefore validated the micro-array results by real-time PCR of this gene and normalized NOXA expression to the expression of the house-keeping gene hypoxanthine phosphoribosyltransferase (HPRT). Indeed, NOXA RNA levels rose slightly in early HF1 cells, but was markedly reduced in late HF1 cells (Figure 3B). Integration of the micro-array data and real-time PCR results show a marked reduction in its basal activity in late HF1 cells, but not in early HF1 cells.

**Table 1:** Expression of p53 targets

GENE	Primary ker	Early HF1	Late HF1
GADD45a	1±0.035	0.86±0.074	0.29±0.112
P21	1±0.030	0.77±0.151	0.13±0.151
NOXA	1±0.017	1.47±0.288	0.13±0.012
MMP2	1±0.45	0.36±0.092	0.46±0.044
IGFBP3	1±0.042	1.25±0.120	0.63±0.277
TGFalpha	1±0.183	0.60±0.230	0.14±0.051
GAPDH	1±0.114	1.08±0.142	1.12±0.100

Table1: Expression of p53 targets according to Affymetrix Human Genome U133A microarrays. Results were normalized to the expression in the primary keratinocytes. GAPDH expression is given as internal control. Standard deviations are of three arrays for each cell type.

Due to the possible involvement of p53 in the stress response, we examined the changes in p53 activity after serum starvation. We used NOXA as a marker for pro-apoptotic p53 activity. Surprisingly, real-time PCR of NOXA showed that although the basal NOXA RNA level was reduced, induction in response to starvation increased as transformation proceeded (Figure 3C). We confirmed these results with a reporter assay, where firefly-luciferase (FFL) was expressed under the control of p53-response elements, with mutant response elements serving as controls. Differences in transfection efficiency were eliminated by normalization to renilla-luciferase under the control of the CMV-promoter. FFL activity was measured under basal growth conditions and following 24 h starvation. FFL expression was not induced by starvation in the primary keratinocytes, but was induced slightly in early HF1 cells, and reached 5-fold induction in late HF1 cells (Figure 3D). Thus, using both real-time PCR and the reporter assay, we saw a direct correlation between the stimulation of p53 pro-apoptotic activity during serum starvation and the induction of apoptosis.

### Anti-apoptotic signaling is reduced in early stages of transformation

**Figure 4:**
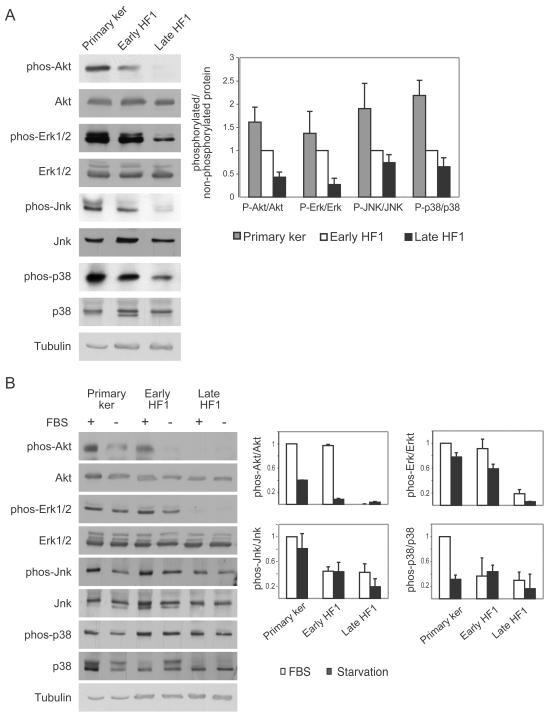
Anti apoptotic signaling is reduced both under basal conditions and under stress. A. Representative western blots of PKB/Akt, Erk, Jnk and p38 in their phosphorylated and non-phosphorylated forms under normal growth conditions. Blots were reacted with the indicated antibodies as described in “Experimental Procedures”. Tubulin serves as a loading control. Graph shows quantification of phosphorylated protein normalized to the total protein level. Results were normalized to the levels in early HF1 cells. Error bars are standard errors of three independent experiments. B. Representative western blots of cells under normal growth conditions and after 24 h starvation. Blots were reacted as in A. Graphs show quantification of protein phosphorylation level vs. total protein level. Results were normalized to the protein level in untreated keratinocytes. Error bars are standard errors of three independent experiments.

Given the increased apoptosis in late HF1 cells and increased activation of p53 under starvation, we examined the activity of key anti-apoptotic signaling pathways in the cells. The phosphorylation of PKB/Akt and Erk was gradually reduced as a function of the degree of transformation (Figure 4A). We also examined the status of the stress kinases Jnk and p38, which act as pro- or anti-apoptotic proteins in different conditions and cellular systems. Phosphorylation of both Jnk and p38 was also reduced with transformation (Figure 4A). Total protein levels of PKB/Akt, Erk, Jnk and p38 remained almost unchanged.

We investigated the response of the PKB/Akt, Erk, Jnk and p38 proteins to serum starvation. Twenty-four hour starvation induced a reduction in PKB/Akt phosphorylation most dramatically in the primary keratinocytes and in early HF1 cells, with no effect on the level of PKB/Akt protein (Figure 4B). Even after starvation, the phosphorylated PKB/Akt level was higher in the primary keratinocytes than in unstarved late HF1 cells and could still transmit anti-apoptotic signals. Less profound effects were seen on the phosphorylation of Erk, Jnk and p38 in response to starvation (Figure 4B). We showed that in late HF1 cells basal activity of the anti-apoptotic proteins: PKB/Akt, Erk, Jnk and p38 are very low compared to the normal counterparts. Their activities are not induced upon serum starvation, therefore do not enable survival under stress.

### IGF1 rescues HF1 cells from serum starvation

**Figure 5:**
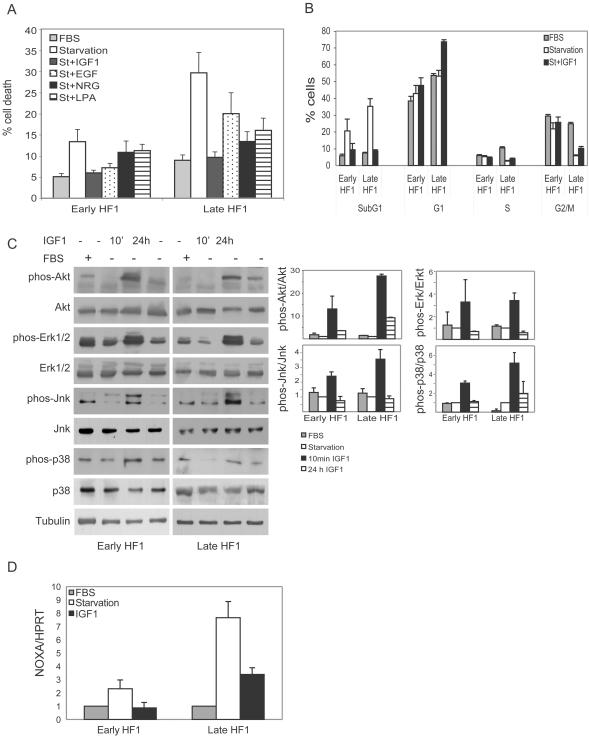
IGF1 rescues HF1 cells from serum starvation, activates PKB/Akt, Erk, Jnk and p38, and inactivates p53. A. Early and late HF1 cells were serum starved for 48 h in the presence of 50 ng/ml IGF1, 30 ng/ml EGF, 30 ng/ml NRG or 10 µM LPA. Stimulated cells were compared to starved and non-starved (FBS) cells. Cells were fixed, stained with propidium iodide and analyzed by flow cytometry. The sub-G1 fraction was quantified as a measure of the apoptotic cells. Error bars represent the standard error of at least three independent experiments. B. Cell cycle analysis of non-starved and starved cells in the presence or absence of IGF1. After 48 h treatment cells were fixed, stained with propidium iodide and analyzed by flow cytometry. Error bars represent the standard error of at least three independent experiments. C. Representative western blots of cells after IGF1 stimulation. HF1 cells were starved for 24 h and stimulated with 50 ng/ml IGF for the last 10 minutes or the whole 24 h of treatment. Stimulated cells were compared to starved and non-starved cells. Blots were reacted with the indicated antibodies. On the right, the densities of the phosphorylated proteins were normalized to the levels of the “total” proteins (phosphorylated and non-phosphorylated), where the starved cells were assigned a value of 1. Tubulin serves as a loading control. Error bars are standard errors of three independent experiments. D. Real-time PCR results of NOXA expression vs. HPRT expression in cells under normal condition or under starvation with or without IGF1. Results were normalized to the results from untreated controls of each cell. Error bars are standard errors of three independent experiments.

We investigated whether sensitivity to stress results from the low anti-apoptotic activity seen in late HF1 cells, and whether these signaling changes are responsible for p53 activation. Due to very low transfection efficiency, and high toxicity of the transfection reagents, we could not introduce dominant-negative constructs or siRNA into the cells. We therefore used a pharmacological approach. First we looked for factors that would rescue the cells from apoptosis when added to the starvation medium and examined the effects of selective pharmacological agents on their action. HF1 cells were starved for 48 h in the presence of insulin-like growth factor 1 (IGF1), epidermal growth factor (EGF), neuregulin (NRG), or lysophosphatidic acid (LPA). The first three are major factors involved in epithelial cell growth and survival [20]–[22], and stimulate known anti-apoptotic pathways. LPA is a major component of serum, and is very often responsible for its effects [23]. We examined cell death by propidium iodide staining and quantification of the sub-G1 fraction of the cell cycle. All four factors reduced the fraction of apoptotic cells. Early HF1 cells were rescued completely by both IGF1 and EGF, while NRG and LPA had smaller effects. Late HF1 cells were rescued completely by IGF1, while EGF, NRG and LPA had weaker effects (Figure 5A). Clearly IGF1 was the most potent factor for the rescue from starvation in both cell types. Furthermore, as the cells became more transformed, the significance of the other factors investigated was reduced.

Indeed treatment of HF1 cells with 50 ng/ml IGF1, during starvation, completely rescued cells from apoptosis. IGF1 treatment reduced the sub-G1 fraction of cells to the basal state in both early and late HF1 cells (Figure 5B). Cell cycle analysis after 48 h starvation with or without IGF1 showed that starvation increased the sub-G1 fraction, accompanied by a reduction in the fraction of cells in the S and G2/M phases, mainly in late HF1 cells. Rescue by IGF1 reduced the sub-G1 fraction in both cell types, accompanied by an increase in the fraction of cells in G1. IGF1 did not change the fraction of cells in S phase and had only a minor effect on cells in G2/M. Under these conditions, IGF1 transmitted a strong anti-apoptotic signal for survival, but did not suffice for cell cycle progression. Thus the cells became dependent on a single agent, IGF1, for survival under serum starvation.

### IGF1 rescues HF1 cells via activation of PKB/Akt and JNK and inactivation of p53

IGF1 is an upstream regulator of: PKB/Akt, Erk, Jnk, p38 and p53 [21], [24]–[28]. We therefore explored which of these proteins mediate survival of HF1 cells under stress. To determine which pathway downstream of the IGF1R is responsible for the rescuing activity of IGF1, we first studied which signaling proteins are stimulated by IGF1. We serum starved the cells for 24 h and added IGF1 either during the whole starvation period or during the last 10 min before cell lysis (Figure 5C). We looked at the phosphorylation of the above-mentioned IGF1R targets: PKB/Akt, Erk, Jnk and p38. PKB/Akt was phosphorylated by IGF1, after both short and long stimulations. PKB/Akt phosphorylation was very high following the short stimulation, but even after 24 h stimulation the phosphorylation level of PKB/Akt was at least as high as the level in the presence of 10% serum. Erk, Jnk and p38 were activated only after short stimulation with IGF1, but might nevertheless have initiated long-term effects. P53 activity, as detected by NOXA expression was down-regulated by IGF1 (Figure 5D).

**Figure 6:**
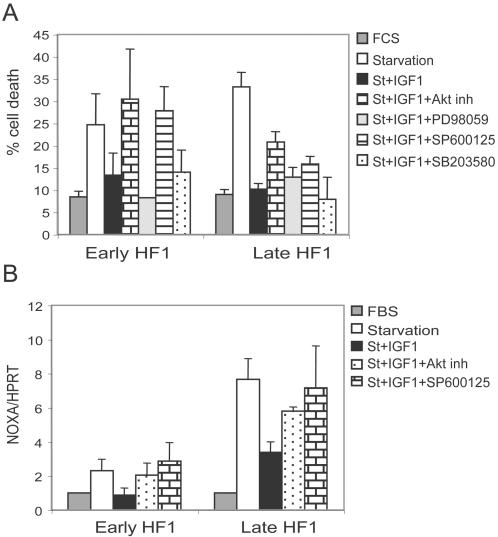
PKB/Akt and Jnk mediate IGF1 rescue of cells and the inactivation of p53 by IGF1. A. Cells were starved for 48 h in the presence of IGF1 and treated with either 10 µM Akt inhibitor, 50 µM PD98059, 10 µM SP600125 or 1 µM SB203580. Control cells were non-starved and starved cells with or without IGF1 treatment. Cells were fixed, stained with propidium iodide and analyzed by flow cytometry. The sub-G1 fraction represents cell death. Error bars represent standard error of three independent experiments. B. Real-time PCR results of NOXA vs. HPRT of cells under normal conditions or during starvation with or without IGF1 in the presence or absence of 10 µM Akt inhibitor or 10 µM SP600125. Results were normalized to the results from untreated controls of each cell. Error bars are standard errors of two independent experiments.

We next examined the effects of specific kinase inhibitors on IGF1-mediated rescue from starvation. In early HF1 cells, treatment with the PKB/Akt inhibitor or the JNK inhibitor SP600125, abrogated the IGF1 effect completely (Figure 6A), suggesting that PKB/Akt and Jnk are responsible for the IGF1R-mediated rescue. In late HF1 cells, again PKB/Akt inhibition and Jnk inhibition showed the strongest effects, but only partially reversed IGF1 rescue. In contrast, the Erk inhibitor PD98059 and the p38 inhibitor SB203580 had no effect on cell survival. Thus IGF1 induced its anti-apoptotic activity mainly via activation of PKB/Akt and Jnk.

We then investigated whether the effect of IGF1 on p53 is mediated through PKB/Akt and Jnk. We serum starved the cells in the presence of IGF1 and the PKB/Akt inhibitor or the Jnk inhibitor SP600125. Under these conditions, p53 activity (as judged by real-time PCR of NOXA) was markedly elevated compared to the IGF1 treated cells (Figure 6B).

### Loss of robustness and dependence on IGF1 is detected also in HPKII cells, HPV16 immortalized keratinocytes, but not in the cervical cancer cell-line, SiHa

**Figure 7:**
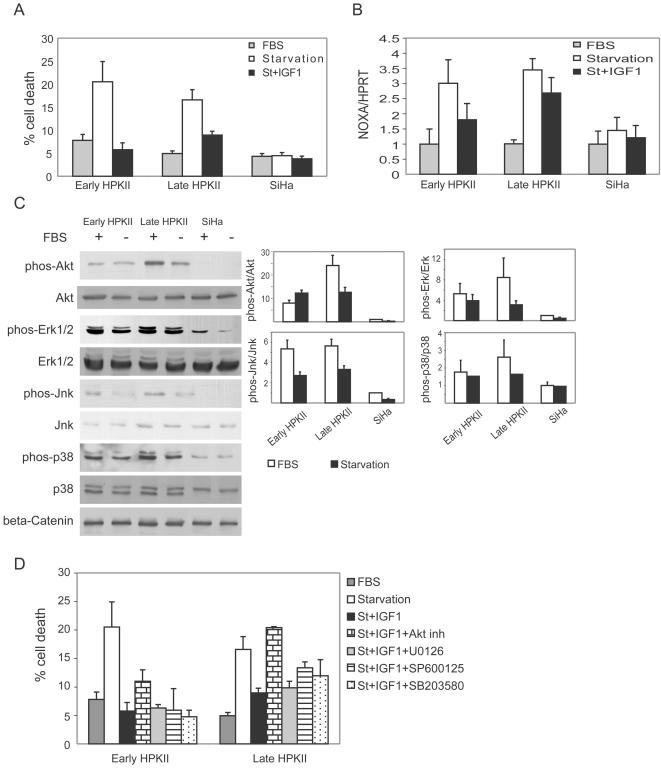
HPKII cells, but not SiHa cells, are sensitive to starvation, due to p53 induction in the absence of IGF1. A. Cells were starved for 48 h with or without IGF1. Cells were fixed, stained with propidium iodide and analyzed by flow cytometry. The subG1 fraction represents cell death. B. Real-time PCR results of NOXA vs. HPRT of cells under normal conditions or during starvation with or without IGF1. C. Representative western blots of PKB/Akt, Erk, Jnk and p38 in their phosphorylated and non-phosphorylated forms under normal growth conditions and upon 24 h starvation. Reaction with anti-beta-Catenin serves as a loading control. Blots were reacted with the indicated antibodies as described in “Experimental Procedures”. Graph shows quantification of phosphorylated protein normalized to the total protein level. Results were normalized to the levels in untreated SiHa cells. Error bars are standard errors of three independent experiments. D. Cells were starved for 48 h with or without IGF1 and treated with either 10 µM Akt inhibitor, 10 µM U0126, 10 µM SP600125 or 1 µM SB203580. Cell death analysis was performed as in A. Error bars represent standard error of four experiments.

We investigated another system of HPV16 immortalized keratinocytes, HPKII cells from early stages (∼70 doublings) and late stages (∼300 doublings). HPKII cells from early and late passages underwent ∼20% apoptosis in response to 48 h of starvation (Figure 7A). Like HF1 cells, HPKII cells were rescued from serum starvation by IGF1. Early HPKII cells were fully rescued by IGF1, while in late HPKII cells, IGF1 induced 60% rescue (Figure 7A). Furthermore, we found a clear correlation between induction of apoptosis and p53 induction. In both early and late passage HPKII cells, p53 was induced ∼3.5 fold in response to 24 h starvation, and IGF1 reduced the induction of p53 (Figure 7B). In early HPKII cells, where IGF1 fully rescued cells from starvation, IGF1 reduced p53 activity by 70%, whereas in late HPKII cells, where IGF1 induced 60% rescue from starvation, it reduced p53 by 40%. Thus, starvation effects both HPKII and HF1 cells in a similar manner, both with regard to their p53 response and to the attenuating effects of IGF1.

Unlike HF1 cells, which showed a progressive decrease in phosphorylation levels of the anti-apoptotic proteins PKB/Akt, Erk, Jnk and p38, these were elevated in early HPKII cells, and were higher in late HPKII cells (Figure 7C). Like HF1 cells, starvation led to a significant decrease in the phosphorylation of these proteins, mainly in late HPKII cells.

In order to find the anti-apoptotic protein through which IGF1 rescues HPKII cells, we starved the cells in the presence of IGF1 and in the presence kinase inhibitors. Inhibition of Akt totally reversed the rescue effect of IGF1 in late HPKII cells, whereas it only partially reversed the effect of IGF1 in early HPKII cells (Figure 7D). The MEK1 inhibitor, U0126, Jnk inhibitor, SP600125 and p38 inhibitor, SB203580 had weaker effects on cell death. Thus rescue of HPKII cells depends mainly on PKB/Akt activation whereas rescue of HF1 cells requires both PKB/Akt and Jnk.

Investigation of the cervical cancer cell line SiHa showed that unlike the HPV16-immortalized cells, fully transformed cells were not sensitive to serum starvation (Figure 7A), and p53 was not activated under these conditions (Figure 7B). But, like in late HF1 cells, in SiHa cells anti-apoptotic signals of PKB/Akt, Erk, Jnk and p38 were very low under normal growth conditions and upon starvation (Figure 7C).

These data show that sensitivity to stress increases due to activation of p53 in two independent cell lines in which transformation was initiated with HPV16. The early transformed cells depend largely or exclusively on PKB/Akt activity, which inhibits p53 induction, for their survival upon starvation. Presumably, in HPKII cells (as in HF1 cells), the decrease in anti-apoptotic activity upon stress enables p53 induction and apoptosis. In contrast, in SiHa cells, where the activities of these enzymes are even lower than in starved HPKII cells, p53 is not induced and cells survive upon starvation, most likely due to the presence of other anti-apoptotic networks, which have emerged on the path to full transformation.

## Discussion

In an attempt to understand the long process that leads to HPV16-mediated transformation, we have compared primary human keratinocytes and HPV16-immortalized keratinocytes, HF1 and HPKII cells, at various stages of in vitro transformation. Presumably, most of the differences between primary keratinocytes and early passage HF1 and HPKII cells are related to the introduction of the HPV16 genome, and the differences between early passage and late passage HF1 and HPKII cells, result from prolonged passaging in vitro. As the HF1 cells became progressively more transformed their growth rate accelerated and they gained the ability to survive in soft agar (Figure 1). Late passage HF1 and HPKII cells can be considered to be pre-cancerous. Although they did not form detectable tumors in nude mice, HPKII cells did form benign cysts [12], [13]. During the course of transformation, the HPV16-immortalized cells became more prone to cell death upon stress (Figure 2,7). These results are supported by previous findings showing that transfection of cells with the HPV16 E6 gene sensitizes cells to genotoxic stress [15]. E6 expression impairs nucleotide excision repair, reducing the cells' capability to recover from DNA damage [14], and E7 expression reduces survival after UV irradiation [16]. Impairment of cell-cycle control due to Rb and p53 down-regulation would be expected to result in cell death in response to environmental stress. Indeed, early HF1 cells highly express HPV16 genes (Figure 3A) and show higher sensitivity to both serum starvation and Cisplatin compared to normal keratinocytes. In the more transformed late HF1 cells, however, HPV16 gene expression is reduced; therefore their expression cannot be the immediate cause of the even greater sensitivity in these stages.

The interplay between p53 and Rb is very complex and involves the E2F transcription factor family. Rb binds and inhibits the E2Fs, which stabilize and activate p53 by several mechanisms [29]. First, E2F upregulates p14ARF, which reduces Mdm2-induced degradation of p53 [29]. Moreover, it induces transcription of ATM, which phosphorylates and stabilizes p53 [30], and can also upregulate the p53 binding partners, ASPP1/2, JMY, TP53INP1, which elevate p53 pro-apoptotic activity [31]. Here we have shown high E7 expression and low Rb level in early HF1 cells, implying high E2F activity. E2F activation of p53 may counteract E6 activity, explaining why p53 was only slightly affected in early HF1 cells. In contrast, in late HF1 cells we found high Rb levels, which may have led to lower E2F activity, and hence to the very low basal activity of p53 in these cells.

The reduction in HPV16 gene expression in late HF1 cells is most probably caused by changes in cellular signaling components. HPV16 gene expression has been shown to be controlled by cellular factors such as AP1, NF1 and glucocorticoids, which bind the P97 HPV promoter [32], [33]. We show that in late HF1 cells MAP kinase activity is decreased compared to early HF1 cells (Figure 4A). Reduced MAP kinase activity is expected to cause reduced AP1 activity, leading to the observed reduction in HPV expression, and the rise in Rb level in late HF1 cells. Indeed, results from our laboratory show AP1 activity is markedly reduced in late HF1 cells (Kravchenko-Balasha et al, in preparation). Changes in Rb level during cervical cancer development have been documented by Salcedo et al [34]. These authors showed that Rb levels were low in HPV16 infected, pathologically normal epithelium, but rose in squamous intraepithelial lesions (SIL), a pre-cancerous state. Rb expression was then reduced in invasive cervical cancer. Possibly late HF1 cells represent a stage similar to SIL, an intermediate stage of transformation.

The amount of p53 was only slightly reduced with transformation, but there was a marked difference in its activity and electrophoretic mobility, suggesting different post-translational modifications of the protein. The activity of p53 was markedly reduced in late HF1, cells as assessed by the transcription of its targets. We examined p53 induction both by real-time PCR of NOXA and by reporter assay. Surprisingly, we found that p53 induction in response to stress increased with transformation, even though basal p53 activity was very low in the more transformed cells. While p53 activity was not induced at all by stress in primary keratinocytes, and was only slightly induced in early HF1 cells, there was a 5–7 fold induction in late HF1 cells (Figure 3C,D) and 3.5 fold induction in HPKII cells (Figure 7B). In fully transformed SiHa cells, we detected no activation of p53 upon starvation, neither was apoptosis induced. It has been shown that SiHa cells have very high Stat3 activity [35], which may prevent p53 induction [36], and account for the resistance of SiHa cells to stress. These cells also highly express the anti-apoptotic BH3 protein MCL1 [37]. Thus, it seems that SiHa cells represent the transformed state beyond the addictive stage, when robustness has been re-established. It has been reported that p53 can be activated in these cells in response to adriamycin treatment, but not in response to oxidative stress by hydrogen peroxide [38]. Presumably the differential response to various stresses and various stress intensities, results in disparity in p53 activation, which leads to differential cell fate.

Little is known about p53 induction in normal epithelial cells. Girinsky et al [39] showed that p53 is highly induced in response to stress in normal stromal cells, but they detected no induction in normal epithelial cells. The reasons for these differences are still unknown, but most probably reflect the robustness of the normal keratinocyte. Perhaps, the lack of p53 activation in normal epithelial cells increases their resistance to various external stresses, and may be essential for epithelial tissue integrity.

The increased apoptosis in late HF1 cells, in conjunction with the increase in stress-induced p53 pro-apoptotic activity, led us to investigate anti-apoptotic signaling. We detected a decrease in the activity of key anti-apoptotic signaling components in HF1 cells. The phosphorylation of PKB/Akt and Erk, Jnk and p38 was gradually reduced as a function of the degree of transformation (Figure 4A), while total protein levels remained almost constant. Other studies have shown high activities of Akt and Erk in fully transformed cancer cells [40], [41]. Many cancer models were developed by introduction of active oncogenes into primary or immortalized cells [42]. In these models it is expected that anti-apoptotic proteins would be activated by the introduced oncogenes even during early stages. In contrast, our model mimics the physiological situation, in which normal cells are infected with HPV16, and the cells replicate for years and accumulate mutations. The phenomenon of decreased anti-apoptotic signaling during cancer development is supported by our results from SiHa cells, which show low activities of PKB/Akt, Erk, Jnk and p38. We propose that reduced anti-apoptotic activities of the above-mentioned proteins are a common feature in transformation, where in both HF1 and HPKII PKB seems to play a key role, where in the latter is more prominent.

During stress, anti-apoptotic proteins are often activated by several autocrine pathways, and enable cell survival [43], [44]. Full blown cancer cells utilize growth factors such as IGF1 to overcome stress or pro-apoptotic signals, by re-igniting the downstream pathways. In HF1 cells, phosphorylation of PKB/Akt, Erk, Jnk and p38 was reduced during serum starvation (Figure 4B). In HPKII cells, although we did not detect a similar reduction in anti-apoptotic activities under normal growth conditions, we did see that the activities of PKB/Akt, Erk, Jnk and p38 are mostly reduced upon starvation (Figure 7C). Nonetheless, the inactivation of these growth-promoting anti-apoptotic proteins during starvation did not enable survival of the transformed cells during stress. Apparently, in late stage HF1 and HPKII cells we “capture” the transformation process at a stage just prior to the onset of autocrine activation characteristic of fully neoplastic cells. Namely at this stage cells are hyper-sensitive to stress (see also ref. 9).

We found a correlation between p53 induction, decreased anti-apoptotic signaling upon starvation and sensitivity to stress. To confirm the connection between these factors we tried to activate the anti-apoptotic proteins, or inactivate p53 in early and late HF1 cells, and reverse the effect. We were able to rescue the cells from apoptosis by adding IGF1 to the starvation medium. As transformation proceeds cell dependence on IGF1 increased. Figure 5A shows that late HF1 cells are fully rescued only by IGF1, while early HF1 cells could also be rescued by EGF, implying that the late HF1 cells had become addicted to IGF1. Cervical cancer cells have been shown to become dependent on IGF1R activity for their growth and survival [45]–[47] . It has also been shown that in early stages of transformation cells depend on external IGF1, secreted from stromal cells, while fully transformed cancer cells secrete IGF1, and their survival depends on this autocrine pathway [43]. Here, IGF1 induced a strong anti-apoptotic signal rather than a proliferative signal. IGF1 treatment enabled cell survival during starvation, but did not enable cell cycle progression in the absence of nutrients, and therefore treatment resulted in arrest in G1 phase of the cell cycle. Thus, our data suggest that the property of cancer cells being addicted to a particular pathway [48] develops in the precancerous stage.

We found that PKB/Akt and Jnk activities were up-regulated and p53 activity down-regulated by IGF1 (Figure 5D). Accordingly, Inhibition of PKB/Akt or Jnk (Figure 6A) led to activation of p53 (Figure 6B), and therefore abrogation of the IGF1 rescuing activity. We conclude that during stress, low activity of PKB/Akt and Jnk enables p53 activation and apoptosis in late HF1 cells. The importance of PKB/Akt in mediating the rescue effect of IGF1 was shown here also in HPKII cells.

IGF1 and EGF very often transmit their signal through similar down-stream signaling pathways. One possible explanation for the higher potency of IGF1 in the rescue of HF1 cells may come from different levels of their receptors. Another explanation may come from their effects on the IGF binding protein 3 (IGFBP3). It has been shown that p53 induces transcription of IGFBP3 [49]. Moreover, IGFBP3 was induced by serum starvation, and induction was inhibited by EGF [50]. In our system both EGF and IGF1 may down-regulate p53, possibly leading to decreased expression of IGFBP3. Exogenous IGF1, but not EGF might surmount the low levels of IGFBP3, leading to rescue from starvation.

In summary, using an in vitro model of keratinocyte transformation by HPV16, we have shown that during the course of transformation, cells develop increased sensitivity to stress, which correlates with higher activation of p53 and reductions in the activities of PKB/Akt, Erk, Jnk and p38 in HF1 cells. We show that the low activities of PKB/Akt and Jnk are responsible for the increased cell death of late HF1 cells, and that their low activities during stress enable p53 induction and initiation of apoptosis, when deprived of IGF1, and nutrients. The reduction in the anti-apoptotic activity reduces the robustness of the transformed cells and results in more cell death. We show that as transformation progresses cells become more dependent on IGF1 for their survival. Normal epithelial tissue seems to be resistant to damaging agents, most probably because of the robustness of the anti-apoptotic network, and the quiescent p53. During transformation normal tissue integrity and function are impaired, anti-apoptotic signals are down-regulated, leading to hyper-activation of p53 and increased cell death in response to stress. Similar results were obtained with HPKII cells, which were developed independently. Significantly, SiHa cells, which represent fully transformed HPV16 infected cells, are robust and do not succumb to starvation, most likely since they have acquired an anti-apoptotic network in which Stat3 and MCL-1 may play a pivotal role [35], [37] . Our results suggest that on the path of HPV16 induced transformation, cells acquire addiction to IGF1 but, when progressing to the fully malignant phenotype, they overcome this Achilles heel and re-establish robustness, characteristic of cancers refractory to therapy.
